# A novel DEAH-box helicase 37 mutation associated with differences of sex development

**DOI:** 10.3389/fendo.2023.1059159

**Published:** 2023-03-30

**Authors:** Yun Wan, Richeng Yu, Jianhua Luo, Ping Huang, Xingju Zheng, Liqun Sun, Kui Hu

**Affiliations:** ^1^ Department of Endocrinology, Guizhou Provincial People’s Hospital, Guiyang, China; ^2^ Department of Pathology, Guizhou Provincial People’s Hospital, Guiyang, China; ^3^ Department of Medical Imaging, Guizhou Provincial People’s Hospital, Guiyang, China; ^4^ Division of Cardiology, Department of Pediatrics, The Hospital for Sick Children, University of Toronto, Toronto, ON, Canada; ^5^ Department of Cardiovascular Surgery, Guizhou Provincial People’s Hospital, Guiyang, China

**Keywords:** 46, XY differences of sex development, family pedigree, DEAH-box helicase 37, whole-exome sequencing, β-catenin

## Abstract

**Objective:**

To determine the genetic etiology of a family pedigree with two patients affected by differences of sex development (DSD).

**Methods:**

Assess the clinical characteristics of the patients and achieve exome sequencing results and *in vitro* functional studies.

**Results:**

The 15-year-old proband, raised as female, presented with delayed puberty and short stature associated with atypical genitalia. Hormonal profile showed hypergonadotrophic hypogonadism. Imaging studies revealed the absence of a uterus and ovaries. The karyotype confirmed a 46, XY pattern. Her younger brother presented with a micropenis and hypoplastic scrotum with non-palpable testis and hypospadias. Laparoscopic exploration was performed on the younger brother. Streak gonads were found and removed due to the risk of neoplastic transformation. Post-operative histopathology showed the co-existence of Wolffian and Müllerian derivatives. Whole-exome sequencing identified a novel mutation (c.1223C>T, p. Ser408Leu) in the Asp-Glu-Ala-His-box helicase 37 gene, which was found to be deleterious by *in silico* analysis. Segregation analysis of the variant displayed a sex-limited, autosomal dominant, maternal inheritance pattern. *In vitro* experiments revealed that the substitution of 408Ser by Leu caused decreased DHX37 expression both at the mRNA and protein levels. Moreover, the β-catenin protein was upregulated, and the p53 protein was unaltered by mutant *DHX37*.

**Conclusions:**

We described a novel mutation (c.1223C>T, p. Ser408Leu) of the *DHX37* gene associated with a Chinese pedigree consisting of two 46, XY DSD patients. We speculated that the underlying molecular mechanism might involve upregulation of the β-catenin protein.

## 1 Introduction

According to the 2006 Chicago consensus statement, differences of sex development (DSD) can be classified into the following: sex chromosome DSD; 46, XY DSD; and 46, XX DSD (1). The occurrence of 46, XY DSD is primarily a consequence of genetic variants leading to disorders of testicular development or defects in androgen biosynthesis or action (1). According to the degree of testicular differentiation, interruption of the male sex-determination pathway causes a phenotype of gonadal dysgenesis (GD) in 46, XY individuals, including partial and complete forms. Embryonic testicular regression syndrome (ETRS), characterized by atypical genitalia and lack of gonadal tissue on one or both sides, has been considered part of the clinical spectrum of 46, XY GD (2). The incidence of 46, XY DSD is estimated to be 1:20000 births and of 46, XY GD around 1:100000 births (3). However, the incidence of DSD or GD may be underestimated due to the rarity of some of the conditions and lack of definitive clinical diagnosis. Besides complete clinical data, detailed genetic analyses, which have been challenging, are pivotal in the diagnosis of DSD. To date, there are at least 18 genes that have been found to be related to 46, XY GD (4). The pathogenic variants in *SRY* (MIM 480000), *NR5A1* (MIM184757), and *MAP3K1* (MIM 600982) are the three most prevalent causes, in total accounting for 40% of individuals with 46, XY GD (4). Pathogenic variants in other sex-determining genes, such as *SOX9* (MIM 608160), *SOX8* (MIM 605923), *GATA4 (*MIM 600576), *DMRT1* (MIM 602424), *FOG2* (MIM603693), *WT1* (MIM 607102), *DHH* (MIM 605423), *CBX2* (MIM 602770), and *DMRT3* (MIM 614754), are found in a small portion of cases (3, 5–7). Therefore, the etiology of the majority of individuals with DSD remains unclear.

Recent studies have identified pathogenic variants in the DEAH-box RNA helicase *DHX37* as a new cause of 46, XY GD and ETRS (8–11). DHX37 is an ATP-dependent RNA helicase and is required for ribosome biogenesis (12). It has been assumed that mutation in the *DHX37* gene might impair ribosome biogenesis; therefore, DSD associated with defective *DHX37* was supposed to be a new ribosomopathy (13). Other biological functions of DHX37 independent of ribosome biogenesis have also been reported. For example, studies of zebrafish carrying mutant *DHX37* demonstrated that *DHX37* physically interacted with the *GlyR α1*, *α3*, *and α4a* subunits, and in mutants the expression of the above transcripts were decreased. Notably, there was no difference in the amount of 18S and 28S rRNA between the wild-type and mutant zebrafish, indicating little effect on ribosome biogenesis (14). Other evidence included genome-wide CRISPR screens identifying DHX37 as an important regulator of human CD8 T-cell activity (15). McElreavey et al. briefly summarized the published *DHX37* pathogenic variants and tried to demonstrate how these variants caused DSD (13). The most common pathogenic variant is the p.Arg308Gln amino acid change (8–11); however, there is no evidence for certain signaling pathways underlying the pathogenesis of DSD caused by *DHX37* variants. In our study, we identified a novel mutant of *DHX37* (c.1223C>T, p.Ser408Leu), which was associated with 46,XY DSD in a Chinese pedigree. *In silico* modeling predicted that the mutation of c.1223C>T would be deleterious to the DHX37 protein. Notably, we proved that the perturbation of *DHX37* led to the upregulation of the β**-**catenin protein, which might underly the mechanism of DSD caused by defective *DHX37*. Our findings extend the variants associated with DSD and highlight the phenotype spectrum associated with *DHX37*. We also provided evidence that DSD caused by defective *DHX37* may have a link with the activation of the Wnt/β **c**atenin pathway.

## 2 Materials and methods

### 2.1 Subjects

Clinical data of two non-twin siblings affected by 46,XY DSD in a Chinese pedigree were collected. Data collected included gender raised as, age at presentation, gynecological examination, hormone profile (follicle-stimulating hormone, luteinizing hormone, testosterone, and anti-Müllerian hormone), karyotyping, family history of DSD, and consanguinity. Abdominal/inguinal ultrasound or urinary CT was performed where specified. A removed gonad stained with hematoxylin and eosin (HE) for histological analyses was provided by the Department of Pathology in our hospital. Written informed consent was obtained from all family members. The Ethics Committee approved this study, including the chromosomal and molecular biology analyses (Institution Review Board of Guizhou Provincial People’s Hospital [2021(No. 3)].

### 2.2 Whole-exome sequencing, data analyses, and *in silico* prediction

Genomic DNA was extracted from peripheral blood using standard procedures (MagPure Buffy Coat DNA Midi KF Kit, MAGEN). Whole-exome sequencing (WES) of the genomic DNA was performed, and a blood sample of the proband was sequenced with PE100+10 on MGISEQ-2000. The sequenced data was aligned to the human genome reference (hg19) using the BWA (Burrows Wheeler Aligner) Multi-Vision software. After alignment, the output files were performed sequencing coverage and in-depth analyses of the target region, single-nucleotide variants (SNVs), and INDEL calling. GATK software was used to detect SNVs and indels, which were filtered and estimated *via* multiple databases, including HapMap, NCBI dbSNP, 1000 human genome dataset, and a database of 100 Chinese healthy adults. We used the Human Gene Mutation Database (HGMD) to screen mutations reported in the published studies. The pathogenic effect of the variant was predicted by three software programs (Polyphen2, Mutation Taster, and PROVEAN) and assessed under the protocol issued by ACMG (16). The potential pathological variant identified by WES was then validated by Sanger sequencing. To predict the molecular consequences of the variant, the homology models of the wild-type (WT) and mutant DHX37 were generated using SWISS-MODEL with the most suitable model (Seq Identity=100% and coverage: 3-1157). To predict the stability of the protein, the protein stability prediction tool I-Mutant (http://folding.biofold.org/i-mutant/) was used.

### 2.3 *In vitro* functional studies of DHX37 mutant

#### 2.3.1 Construction of plasmids

Complementary DNA (cDNA) encoding WT DHX37 was cloned into the digested pcDNA3.1 vector, producing pcDNA3.1-DHX37-WT. The single mutation (p.S408L) was inserted using a Site-Directed Mutagenesis Kit (Vazyme, China), generating pcDNA3.1-DHX37-S408L. The entire coding sequence of both4plasmids was certified by direct sequencing prior to functional studies.

#### 2.3.2 Cell culture, transfection, and functional analyses

A human Sertoli cell line (iCell-0085a, iCell Bioscience Inc., China) was used for molecular studies. The cells were cultured in a special culture medium for human Sertoli cells (iCell-0085a-001b, iCell Bioscience Inc., China) and passaged with standard procedures. The empty expressing vector, mutant, and WT constructs were transiently transfected into the Sertoli cells using the Lipofectamine 3000 Transfection Reagent (Invitrogen, USA) according to the manufacturer’s protocol. Cell viability was observed with Cell Counting Kit-8 (KGA317, KeyGen Biotech, China). The apoptotic rate of cells was evaluated by flow cytometry (FCM) with Annexin V-FITC/PI kit (AP101-100-kit, MultiSciences Biotech, China). Western blot analysis and quantitative real-time PCR were performed as previously described (17). The primary antibody p53 (1:500) was from Affinity Biosciences (AF0879), β catenin (1:500) was from Servicebio (GB11015), DHX37 (1:500) was from Bioss (bs-14320R), and β-actin (1/2000) and GAPDH, which was used as loading control, were from TransGen Biotech (β-actin:HC201; GAPDH : HC301). The horseradish peroxidase-conjugated secondary antibody (1:2,000) was from Beijing Zhongshan Jinqiao Biological Technology (anti-Rabbit ZB-2301; anti-Mouse: TA-09). Protein band densities were quantified using the Image J program. The primers for qPCR were as follows: β-actin: 5’-TGGCACCCAGCACAATGAA-3’ and 5’-CTAAGTCATAGTCCGCCTAGAAGCA-3’; DHX37:5’- CGGCGCTACAACATCAAGG-3’ and 5’-CTTCTTCCCCGGTAGAACGAG-3’.

#### 2.3.3 Immunofluorescence cell staining

Cultured Sertoli cells were washed three times with PBS and fixed with 4% paraformaldehyde for 15 min at room temperature, followed by permeabilization with 0.1% Triton-X-100 for 20 min. Then, the cells were washed three times with PBS and blocked with 5% BSA for 30 min at 37°C. Cells were incubated with the anti-DHX37 antibody (1:200 dilutions, bs-14320R, Bioss, China) overnight at 4°C, followed by the secondary goat anti-rabbit IgG-Cy3 antibody (1:200 dilutions, AS007, ABclonal, China) incubated for 30 min at 37°C. Nuclear staining of the cells involved using DAPI (KGA215-50, KeyGen Biotech, China) for 3 min at room temperature. Images were captured by fluorescence microscopy (Olympus, Japan).

### 2.4 Statistical analysis

GraphPad 9.0 (Prism, USA) was used for data analysis. Quantitative data with normal distribution was presented as means ± SD from at least three independent experiments. Statistical analysis was performed with the use of a one-way ANOVA followed by multiple comparisons with a *post hoc* Tukey’s test. A p value of less than 0.05 was considered to be statistically significant.

## 3 Results

### 3.1 Clinical characteristics of the pedigree

**Figure 1:**
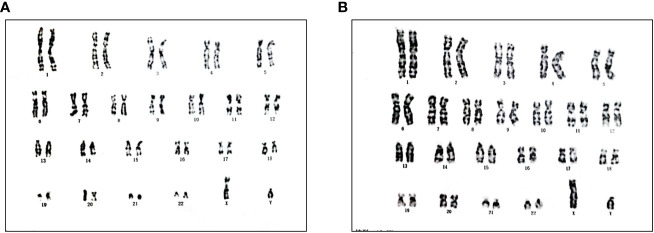
Karyotype of III:1 **(A)** and III:2 **(B)**.

The 15-year-old proband (III:1), raised as female, presented with complaints of short stature, with no signs of puberty and menstrual bleeding. At the time of admission, her height was 139.5cm, and her weight was 34.8kg. She had normal intellectual function and facial appearance, with development of both breasts at Tanner I and absence of pubic and axillary hair. Gynecological examination revealed poorly developed labia, clitoral hypertrophy, and absence of vaginal opening. An abdominal and pelvic ultrasound did not show the ovaries and uterus. Inguinal ultrasound ruled out the presence of testes in the inguinal region. The hormonal profile revealed low levels of both total testosterone (0.4ng/mL, 4.94-32.01ng/ml) and anti-Müllerian hormone (AMH) (0.02ng/ml, 0.96–13.34 ng/mL), but elevated levels of follicle-stimulating hormone (FSH) (73.34 U/L, 0.95-11.95 U/L) and luteinizing hormone (LH) (19.32 U/L, 0.57-12.07 U/L). The karyotype was mapped, demonstrating a 46, XY pattern (**Figure 1A**).

**Figure 2:**
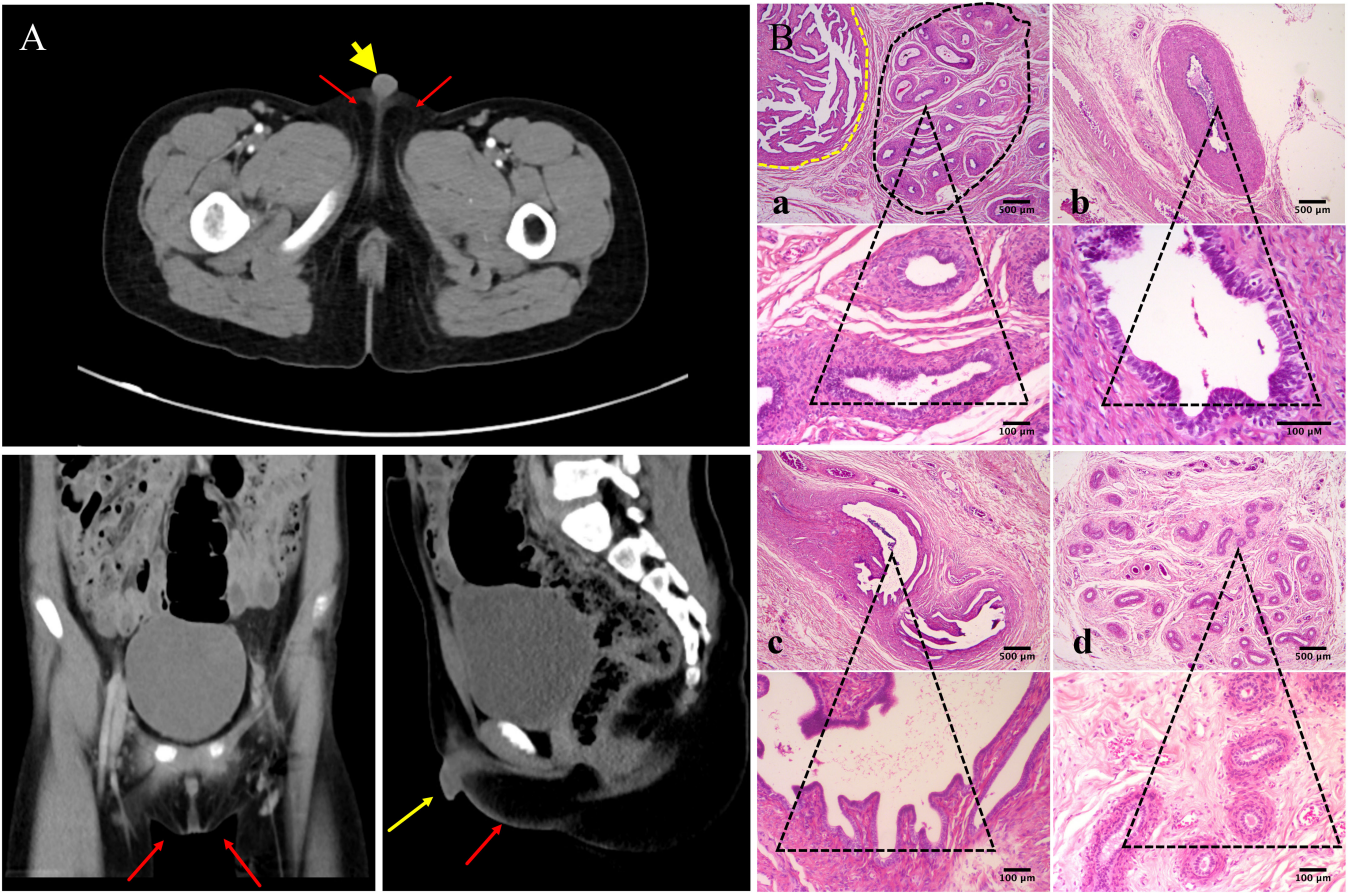
Urinary CT scan and post-surgical histopathology of the gonads of III:2. **(A) ** CT scan of the urinary system shows empty scrotum (red arrows) and small penis (yellow arrow). **(B) ** Histologic analysis of gonad samples from III:2. No gonadal tissue was observed; however, fallopian tube-like and epididymal-like structures were present in both gonads of the patient. **(a)** Fallopian tube-like (circled by yellow dashed line) and epididymal-like structures (circled by black dashed line) on the left side were seen in the same microscopic field; **(b)** remnants of the ductus deferens were also present on the sample from the left side; **(c)** fallopian tube-like and **(d)** epididymis-like structures on the right side. The lower panel shows indicated tissues on the upper panel at higher magnification. Size bars are indicated for each panel. Staining was performed with hematoxylin–eosin.

Further investigation revealed that one of the younger brothers also had 46, XY gonadal dysgenesis. The boy (III:2) was nine years old when admitted to our department. Physical examination revealed a micropenis, hypospadias, and hypoplastic scrotum with non-palpable testis. The hormonal profile revealed a low testosterone level of 0.22 ng/mL (4.94-32.01ng/ml) and a slightly elevated level of FSH 17.27 U/L (0.95-11.95 U/L). The LH level was 1.15 U/L (0.57-12.07 U/L), and the AMH was not examined. The karyotype was 46, XY (**Figure 1B**). A CT scan (**Figure 2A**) of the urinary system showed an empty scrotum and a small penis. Bilateral streak gonads were found upon laparoscopy. In respect of the high risk of gonadal tumors in DSD patients (18), the streak structure was removed, and post-surgical histopathology demonstrated a mixture of epididymis- and fallopian tube-like structures on both sides, as well as remnants of the ductus deferens on the left side (**Figure 2B**). The clinical characteristics of the two affected siblings are listed in **Table 1**.

**Table 1:** Clinical characteristics of III:1 and III:2.

	Gender raised as	Karyotype	Age at presentation	Diagnosis	External genitalia	Internal genitalia	Gonadal histology	LH(U/L) Reference range: 0.57-12.07	FSH(U/L) Reference range: 0.95-11.95	T(ng/ml) Reference range: 4.94-32.01	AMH(ng/ml) Reference range: 0.96-13.34
III:1	Female	XY	15	46,XY TRS	Poorly developed labia, clitoral hypertrophy, absence of vaginal opening	No mullerian structures, no gonads present	NA	19.32	73.34	0.4	0.02
III:2	Male	XY	10	46, XY PGD	Micropenis, hypoplastic scrotum with non-palpable testis, hypospadias	No mullerian structures, no gonads present	Fallopian tube- and epididymis-like structures on both sides, remnants of the ductus deferens on the left side	1.15	17.27	0.22	NA

NA, not available.

We confirmed that the two younger sisters (III:3, III:4) had the 46, XX karyotype (**Supplementary Figures 1A, B**), and the youngest brother had 46, XY (III:5) (**Supplementary Figure 1C**); each of the three children had a normal facial appearance, intellectual development, and age-appropriate development of external genitalia.

### 3.2 Whole-exome sequencing identifies *DHX37* mutation

**Figure 3:**
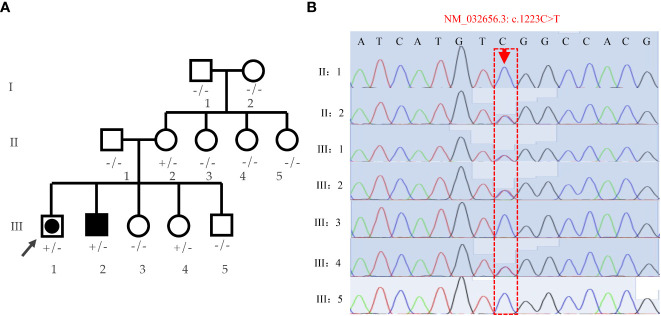
Analyses of the family pedigree with two cases affected by 46,XY DSD. **(A)** Family pedigree. Closed symbols represent affected individuals. The affected male (46, XY males) is indicated by a closed square and the affected individual raised as female (46,XY females) is shown by a large, closed circle within a square. Whole-exome was performed on the proband (indicated by black arrow), and Sanger sequencing were verified on all family members; genotypes are labeled on the chart. +/- heterozygous state; -/- homozygous state for wild-type allele. **(B)** Verification of the S408L mutation by means of Sanger sequencing in the pedigree.

Family pedigree chart is shown in **Figure 3A**. A variant (c.1223C>T; p.Ser408Leu) in exon 9 of the *DHX37* gene was obtained in the sample of the proband by exome sequencing. The mutation was not present in 1000 Genomes, ESP6500, ExAC, GnomAD, and GnomAD-EAS. Three programs (Polyphen-2, Mutation Taster, and PROGEAN) predicted this mutation would be deleterious to the protein function (**Table 2**). The variant was validated by Sanger sequencing in all family members; the representative sequencing results are shown in **Figure 3B**. Both the proband (III:1) and the clinically affected brother (III:2) carried the heterozygous variation. Moreover, their asymptomatic mother (II:2) and one of the younger sisters (III:4) carried the same variation. The rest of the family members (Subjects I:1, I:2, II:1, II:3, II:4, II:5, III:3, and III:5) had no variation.

**Table 2:** Prediction of the pathogenicity of S408L by a range of soft programs.

Program	Score	Prediction
PolyPhen-2	0.999	probably damaging
Mutation taster	0.999999999721836	disease causing
PROVEAN	-5.824	deleterious

**Figure 4:**
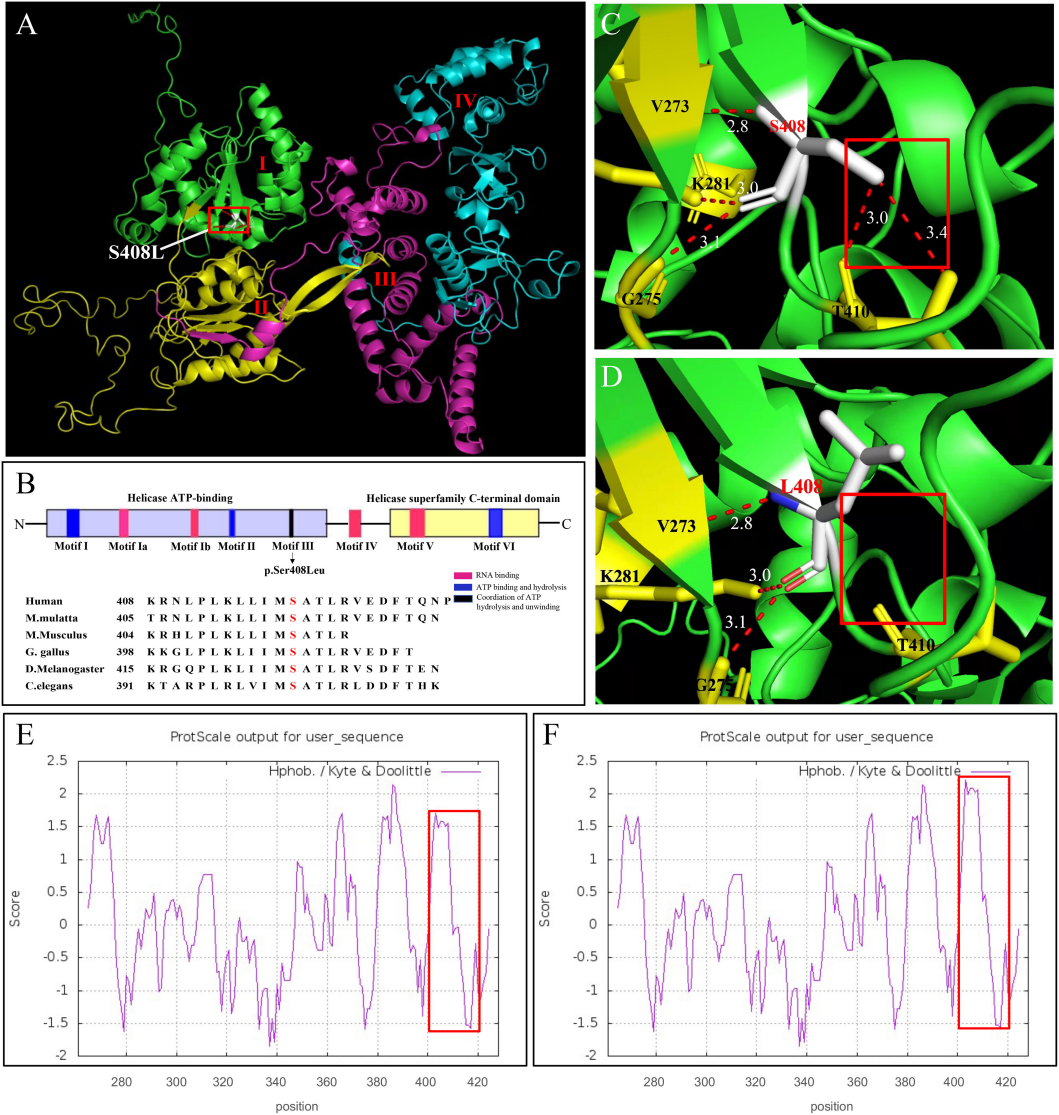
*In silico* modeling of DHX37. **(A)** Functional domains of a homology model of the DHX37 protein. The protein has four functional domains, which are color-coded and labeled. I: ATP-binding DEAH-box helicase (green), II: C-terminal helicase (yellow), III: helicase-associated 2 domain (purple), and oligonucleotide/oligosaccharide-binding-like domain (blue). The variant p.Ser408Leu is located in the helicase ATP-binding domain. **(B)** Schematic protein structure of the helicase core region, i.e., the helicase ATP-binding domain and the helicase superfamily C-terminal domain. Colors represent the main helicase functions. Sequence alignment shows the conservation of the amino acid residue S408 across different species. The variant p.S408L falls within the Motif III, which is implicated in the coordination of ATP hydrolysis and unwinding. A zoomed-in view of protein model with residue S408 **(C)** and L408 **(D)**. S408 and T410 and V273 and G275 were linked by hydrogen bonds in the wild type. After the mutation, the hydrogen bonds between S408 and T410 disappeared. N-, N terminus; -C, C terminus. Kyte and Doolittle hydropathy plot of the DHX37 protein **(E)** before and **(F)** after p.S408L mutation. A score >0 means hydrophobic and <0 means hydrophilic. Higher positive values indicate greater hydrophobicity. The mutation caused increased hydrophobicity in the region between codons 400 and 420, as outlined by red boxes.

We further investigated how the p.Ser408Leu mutation affected the DHX37 protein structure. The DHX37 protein (NP_116045) comprises 1157 amino acids (AAs) and four main domains, including two RecA-like domains, which are the helicase core domains (RecA1: ATP-binding DEAH-box helicase, RecA2: C-terminal helicase), helicase-associated 2 domain (HA2), and oligonucleotide/oligosaccharide-binding fold domain (**Figure 4A**). The conserved motifs of the helicase core region are involved in RNA substrate interaction, ATP binding, and hydrolysis, as well as the coordination of the unwinding activity (9–12). The variant c.1223C>T (p.Ser408Leu) is located in the helicase ATP-binding domain and falls within the Motif III (**Figure 4B**), which has been implicated in the coordination of ATP hydrolysis and unwinding (9–12). Notably, the affected amino acid residue Ser408 is highly conserved across different species (**Figure 4B**), suggesting its structural and functional importance. Homology models of DHX37 generated by SWISS-MODEL showed that Ser408 and Thr410 and Val273 and Gly275 were linked by hydrogen bonds in the wild type (**Figure 4C**). After the mutation, the hydrogen bonds between Ser408 and Thr410 disappeared, and large side chains were introduced (**Figure 4D**). Moreover, as the serine acid is hydrophilic and polar, while the leucine is hydrophobic and non-polar, we also investigated the hydrophobicity of the protein region by use of Kyte and Doolittle hydropathy plots (**Figures 4E, F**); we found that the p.Ser408Leu variant caused the increased hydrophobicity in the region between codons 400 and 420. In this work, we found this mutation increased the stability of the protein by using the protein stability prediction tool, which demonstrated the pathogenic role of the p.Ser408Leu mutant.

### 3.3 *In vitro* functional studies

**Figure 5:**
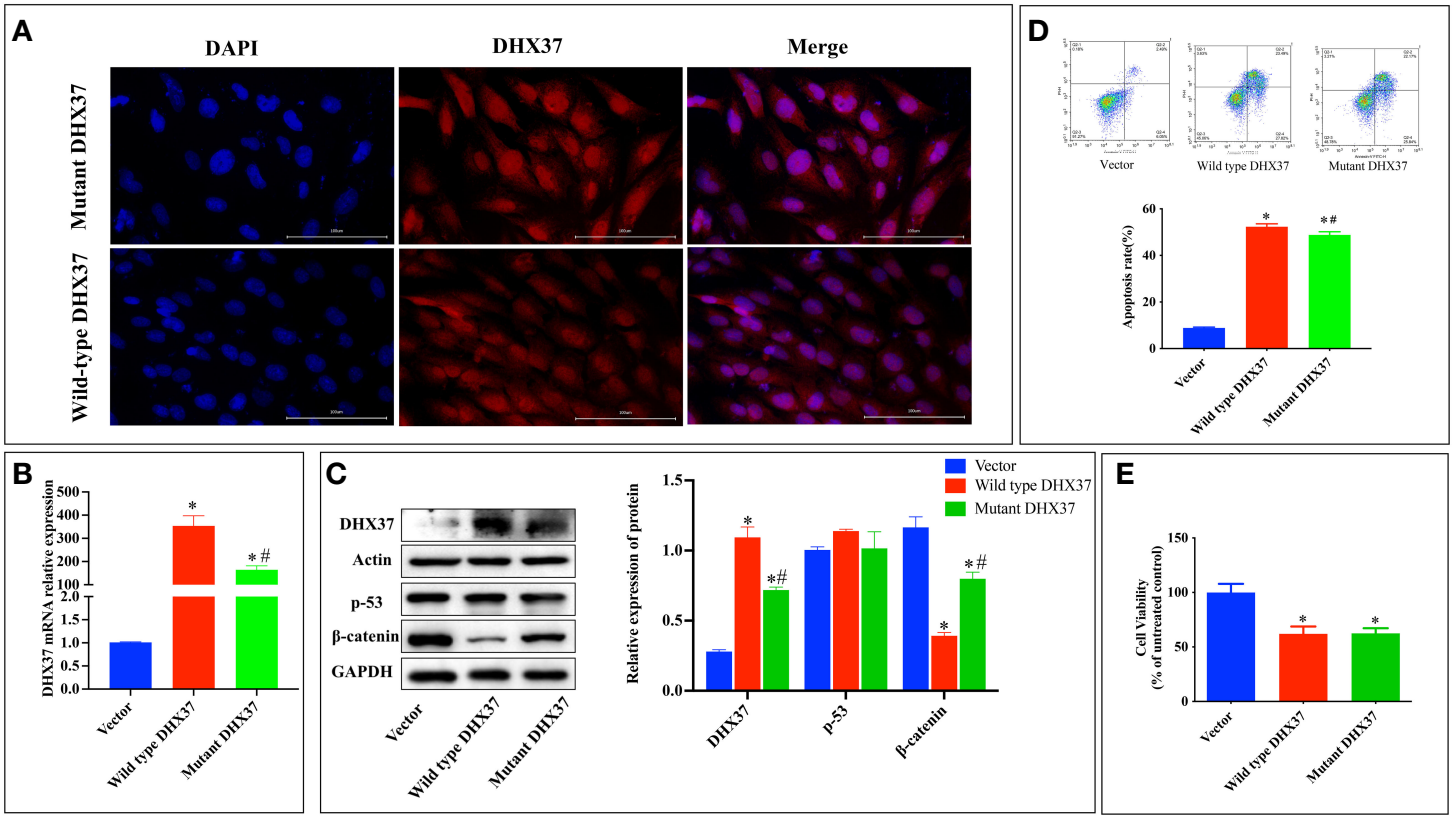
*In vitro* functional studies. **(A)** Cellular localization of mutant and wild-type DHX37 protein. Human Sertoli cells were transfected with wild-type *DHX37* or mutant *DHX37*. Immunofluorescence was performed on the cells using antibodies against DHX37 (red), and the nucleus was stained by DAPI (blue). Scale bars are 100 μm. The highest concentration of the protein was in the nucleus, although protein was also observed in the cytoplasm. Both wild-type and mutant DHX37 exhibited the same cellular localization. **(B)** Relative mRNA expression of *DHX37* when human Sertoli cells were transfected with empty expressing vector, wild-type *DHX37*, and mutant *DHX37*. **(C)** Western blot analysis of DHX37, p53, and the β-catenin protein expression in human Sertoli cells transfected with empty, wild-type, or mutant *DHX37* expression vector. Actin and GAPDH were the housekeeping protein used in this Western blot as a loading control. Effects of the *DHX37* mutation (S408L) on **(D)** apoptosis and **(E)** proliferation of human Sertoli cells transfected with empty, wild-type, or mutant *DHX37* expression vector. Statistical analysis was performed with the use of one-way ANOVA followed by multiple comparisons using a *post hoc* Tukey’s test. *p < 0.05 compared with empty expression vector. #p < 0.05 compared with wild-type *DHX37*.

To further investigate the pathogenesis of the p.Ser408Leu mutation, the pcDNA3.1-WT and pcDNA3.1-S408L plasmids were constructed and separately transfected into human Sertoli cells. To first ascertain the localization of DHX37, we performed immunofluorescence in the Sertoli cells by using an antibody against DHX37. This revealed that DHX37 predominantly localized to nucleoli, although protein was also observed in the cytoplasm. Both wild-type and mutant DHX37 exhibited the same cellular localization (**Figure 5A**). DHX37 expression in cells transfected with either pcDNA3.1-WT or pcDNA3.1-S408L was analyzed by real-time PCR and Western blot. As shown in **Figures 5B, C** compared with the cells transfected with pcDNA3.1-WT, those with pcDNA3.1-S408L displayed decreased DHX37 expression both at the mRNA and protein levels.

Classically, impaired ribosome biogenesis triggers nuclear stress, which leads to cell apoptosis partly through stabilization of the tumor suppressor p53 (19). Moreover, nuclear stress was recently found to activate WNT/β-catenin signaling (20). Therefore, the effect of mutant *DHX37* in p53 and β-catenin signaling was examined. As shown in **Figure 5C**, there is no difference in p53 expression among cells transfected with empty expressing vector, wt-*DHX37*, or mutant *DHX37.* Interestingly, transfection with wt-*DHX37* led to a significant decrease of the β-catenin protein, which was rescued by the mutant *DHX37*. We further examined the effect of DHX37 on cell apoptosis and proliferation. As shown in **Figure 5D**, compared with the cells transfected with empty expressing vector, those with wt-*DHX37* or mutant *DHX37* exhibited an increased apoptosis rate. Furthermore, cells that expressed wt-*DHX37* showed an even higher apoptosis rate than the mutant. As demonstrated in **Figure 5E**, overexpression of both wt-*DHX37* and mutant-*DHX37* decreased cell proliferation, but there was no difference between the two groups.

## 4 Discussion

We identified a heterozygous c.1223C>T mutation (p.Ser408Leu) in exon 9 of *DHX37* in a pedigree affected by 46,XY GD by using WES. The mutation was not present in 1000 Genomes, ESP6500, ExAC, GnomAD, or GnomAD-EAS. Moreover, the p.Ser408Leu substitution is considered probably damaging (0.999) by PolyPhen2, disease-causing (0.999) by Mutation Taster, and has a PROVEAN score of -5.824 (deleterious). In the DEAH-box family of proteins, Ser408 is a highly conserved residue across various species. It falls within Motif III, which is known to couple ATPase and unwinding activity (9–12). The mutation p.Ser408Leu may affect the alignment of the two RecA-like domains responding to NTP binding or fail to assemble the NTP active site responding to nucleic acid binding; therefore, it may impair the coordination of ATP hydrolysis and unwinding (21). We also provided evidence that the p.Ser408Leu variant changed the polarity and stability of the DHX37 protein. Moreover, *in vitro* studies demonstrated that the DHX37 mRNA and protein decreased significantly in cells carrying mutant *DHX37*. Importantly, β-catenin was upregulated by mutant *DHX37*, which may contribute to the pathogenesis of 46, XY DSD caused by defective *DHX37*.

To date, *DHX37* variants associated with 46, XY DSD have ben transmitted either maternally or *de novo*, except for c. G 923A (p. Arg308Gln) (10) and c.C1430T (p. Thr477Met) (9), each of which in a family were reported to be inherited by the proband from their fertile father. In our current study, the *DHX37* variant was delivered from the asymptomatic mother. Moreover, one of the proband’s sisters (III4), who carried the same variant, exhibited a normal phenotype, demonstrating a sex-limited inheritance mode. McElreavey et al. (13) showed that DHX37 expression was higher in male gonads than female, suggesting its important role in regulating the development of male gonads. In our study, with the use of the human Sertoli cell line, we identified the presence of DHX37, which was predominantly localized to the nucleus. The protein was also present in the cytoplasm, consistent with the process of ribosome formation taking place initially in the nucleolus and then in the cytoplasm (13). The expression of DHX37 in human Sertoli cells was also observed by McElreavey et al. (11, 13). However, in another study, while DHX37 was seen in Leydig cell cytoplasm and germ cells at different stages of maturation, rare Sertoli cells displayed a weak and focal cytoplasmatic stain (10). The expression pattern of DHX37 may vary depending on the developmental stages of the testes. In the fetal gonads of mice and humans, DHX37 is expressed only in the somatic cell lineages but not in germ cells (11). In adult human testes, however, the protein is observed in spermatogonia (11). Interestingly, it was found that as the cells differentiated from spermatocytes to spermatids, the protein exhibited a progressive condensation around the nucleus (10). The unique expression pattern of the DHX37 protein may imply its important role in testis development and maintenance of testicular function.

According to previous studies, there is a poor genotype–phenotype correlation in the 46, XY DSD patients associated with *DHX37* variants. Even for those who carry the same pathogenic mutation of DHX37, the genital phenotype can range from predominantly female to male. The degree of virilization depends on the duration of the functional testis before it subsequently vanishes. Although it happens occasionally, there are male carriers who have typical external genitalia and preserved fertility, suggesting sufficient functional testicular tissue for the development of the external genitalia and to support spermatogenesis (9, 10). Unknown genetic modifiers may prevent the appearance of phenotype in individuals with pathogenic *DHX37* variants. In this study, the proband was raised as female with atypical external genitalia and absence of gonads in either side, while her younger brother, who carried the same *DHX37* variant, was raised male with a micropenis and had partially developed internal ducts consisting of a mixture of Wolffian (epididymis-like structures, vas deferens) and Müllerian ducts (fallopian tube-like structures). The proband was clinically diagnosed as 46, XY ETRS, while her younger brother was diagnosed as 46, XY PGD, reinforcing the heterogenicity of 46, XY DSD and that 46, XY GD and ETS form part of the same phenotypic spectrum and share the same etiological mechanism.

Considering the role of DHX37 in ribosome biogenesis, DSD caused by *DHX37* defects is suggested to be a kind of ribosomopathy (13). Exactly how mutant DHX37 proteins cause a highly specific human developmental disorder is confusing, since it is widely expressed and involved in a basic cellular function. McElreavey, K (13, 22). suggested that activation of the canonical WNT/β-catenin pathway may be a possible mechanism. Recent studies revealed that upon nuclear stress challenge, the canonical WNT/β-catenin pathway was transiently activated, followed by p53-dependent apoptosis. It was assumed that activation of the canonical WNT/β-catenin, which regulates a variety of prosurvival processes including cell proliferation and inhibition of cell apoptosis, might serve as a response to sustain nuclear function (20). Notably, inhibition of WNT/β-catenin signaling is necessary for correct testis formation, and stabilization of β-catenin has been identified to cause male-to-female sex reversal in XY gonads (23). With the use of the Sertoli cell line, we demonstrated that overexpression of wt-*DHX37* decreased the expression of the β-catenin protein, which was consistent with the role of DHX37 in the correct formation of testis. However, in the cells transfected with mutant *DHX37* (p.Ser408Leu), the expression of the β-catenin protein was rescued, which may underly the pathogenesis of testicular dysplasia in the current study. Surprisingly, we did not see increases in the p53 protein and cell apoptosis by *DHX37* mutation, which was not supportive of impaired nuclear integrity, raising the possibility that specific biological roles independent of the ribosome formation of DHX37 were involved in the testis development. Future studies may focus on the interaction between DHX37 and β-catenin.

Excision of intra-abdominal gonads is recommended for all XY GD patients, as the risk of germ cell malignancy is as high as 15% ~ 35% (1, 24). Hormone treatment is needed for the induction of puberty, hormone replacement therapy, and suppression of puberty on some occasions based on male/female sex assignment. Overall, in the treatment of DSD, a skilled multidisciplinary group should be involved to facilitate team decisions about assignment/reassignment of male or female sex, surgical issues regarding timing and consent, hormone treatment, and the best possible fertility preservation measures (25). The male patient (III2) was referred to the Pediatric Surgery Department in our hospital for a laparoscopy, and the intra-abdominal gonad bands were removed. Hormone treatment was suggested when it was time for the induction of puberty, usually at age 11–13 in males (25). Surgical exploration was suggested for the proband, since no gonad was found by ultrasound (US). It has been reported that imaging of the gonads by US or MRI is difficult because of the small size and the variable localization in female 46,XY patients (26). Therefore, invasive monitoring is necessary for these patients (18). Unfortunately, after open communication with the patient and her parent, they refused further examination and operation. The patient was upset about her gender identity; therefore, intensive psychological counselling was suggested. If the patient and her parents chose to maintain the female social sex, a low dose of estrogen (one-sixth to one-fourth of an adult dose) was recommended to avoid excessive bone maturation, especially as the patient was concerned about her short stature. Estrogen replacement should be titrated every six months until breast development is complete, after which an adult dose can be maintained continuously (27). Progesterone was not needed in this case, since the patient was found without a uterus (27). However, if the patient chose to change her sex to male, which happens in about 20% of 46,XY DSD patients at a median age of 15 years (28), androgen replacement would be required for masculine pubertal induction.

## 5 Conclusion

We identified a novel mutant of *DHX37* (c.1223C>T, p. Ser408Leu) in a Chinese pedigree affected by DSD. Bioinformatics analysis suggests the variant is pathogenic, consistent with the *in vitro* study that shows the mutation leads to decreased DHX37 expression both at the mRNA and protein levels. Importantly, in our study, the mutant *DHX37* increases the β-catenin protein, which may be responsible for the disturbance of testis development. Our findings extend the variants associated with DSD and increase the phenotype spectrum associated with DHX37. We also highlight the early diagnosis of 46, XY GD with the use of genetic analysis regarding the high risk of developing gonadal tumors, especially in 46, XY GD females. A definitive genetic diagnosis would be beneficial for screening family members and identifying patients with atypical clinical features, along with prenatal genetic counselling for parents preparing to start a family.

## Data availability statement

The sequencing data presented in the study are deposited in the GenBank, accession number OP599354.

## Ethics statement

The Ethics Committee approved this study including chromosomal and molecular biology analyses (Institution Review Board of Guizhou Provincial People’s Hospital [2021(No. 3)]. Written informed consent to participate in this study was provided by the participants’ legal guardian/next of kin. Written informed consent was obtained from the individual(s), and minor(s)’ legal guardian/next of kin, for the publication of any potentially identifiable images or data included in this article.

## Author contributions

KH, LS, and YW designed this project. KH, YW, RY, JL, PH, and XZ participated in the clinical management and data collection. YW and KH organized the genetic analysis. YW, RY, PH, XZ, LS, and KH prepared the manuscript. KH and LS supervised the study and worked on the editing. All authors have read and approved the published version of the manuscript.

## Supplementary material

Supplementary Figure 1 Karyotype of III:3 **(A)**, III:4 **(B)**, and III:5 **(C)**.

## References

[1] Lee PA Houk CP Ahmed SF Hughes IA. Consensus statement on management of intersex disorders. International consensus conference on intersex. Pediatrics (2006) 118(2):e488–500. doi: 10.1542/peds.2006-0738 16882788

[2] Marcantonio SM Fechner PY Migeon CJ Perlman EJ Berkovitz GD. Embryonic testicular regression sequence: A part of the clinical spectrum of 46,XY gonadal dysgenesis. Am J Med Genet (1994) 49(1):1–5. doi: 10.1002/ajmg.1320490102 8172233

[3] Bashamboo A McElreavey K. Mechanism of sex determination in humans: Insights from disorders of sex development. Sex Dev (2016) 10(5-6):313–25. doi: 10.1159/000452637 27915330

[4] Eggers S Sadedin S van den Bergen JA Robevska G Ohnesorg T Hewitt J. Disorders of sex development: Insights from targeted gene sequencing of a large international patient cohort. Genome Biol (2016) 17(1):243. doi: 10.1186/s13059-016-1105-y 27899157 PMC5126855

[5] Baxter RM Arboleda VA Lee H Barseghyan H Adam MP Fechner PY. Exome sequencing for the diagnosis of 46,XY disorders of sex development. J Clin Endocrinol Metab (2015) 100(2):E333–44. doi: 10.1210/jc.2014-2605 PMC4318895 25383892

[6] Portnoi MF Dumargne MC Rojo S Witchel SF Duncan AJ Eozenou C. Mutations involving the SRY-related gene SOX8 are associated with a spectrum of human reproductive anomalies. Hum Mol Genet (2018) 27(7):1228–40. doi: 10.1093/hmg/ddy037 PMC6159538 29373757

[7] Tsai CL Tsai CN Lee YS Wang HS Lee LY Lin CY. Genetic analysis of a Taiwanese family identifies a DMRT3-OAS3 interaction that is involved in human sexual differentiation through the regulation of ESR1 expression. Fertil Steril (2020) 114(1):133–43. doi: 10.1016/j.fertnstert.2020.03.008 32553473

[8] Buonocore F Clifford-Mobley O King TFJ Striglioni N Man E Suntharalingham JP. Next-generation sequencing reveals novel genetic variants (SRY, DMRT1, NR5A1, DHH, DHX37) in adults with 46,XY DSD. J Endocr Soc (2019) 3(12):2341–60. doi: 10.1210/js.2019-00306 PMC6855215 31745530

[9] Zidoune H Martinerie L Tan DS Askari M Rezgoune D Ladjouze A. Expanding DSD phenotypes associated with variants in the DEAH-box RNA helicase DHX37. Sex Dev (2021) 15(4):244–52. doi: 10.1159/000515924 34293745

[10] da Silva TE Gomes NL Lerario AM Keegan CE Nishi MY Carvalho FM. Genetic evidence of the association of DEAH-box helicase 37 defects with 46,XY gonadal dysgenesis spectrum. J Clin Endocrinol Metab (2019) 104(12):5923–34. doi: 10.1210/jc.2019-00984 31287541

[11] McElreavey K Jorgensen A Eozenou C Merel T Bignon-Topalovic J Tan DS. Pathogenic variants in the DEAH-box RNA helicase DHX37 are a frequent cause of 46,XY gonadal dysgenesis and 46,XY testicular regression syndrome. Genet Med (2020) 22(1):150–9. doi: 10.1038/s41436-019-0606-y PMC6944638 31337883

[12] Tanner NK Linder P. DExD/H box RNA helicases: From generic motors to specific dissociation functions. Mol Cell (2001) 8(2):251–62. doi: 10.1016/S1097-2765(01)00329-X 11545728

[13] McElreavey K Pailhoux E Bashamboo A. DHX37 and 46,XY DSD: A new ribosomopathy? Sex Dev (2022) p:1–13. doi: 10.1159/000522004 35835064

[14] Hirata H Ogino K Yamada K Leacock S Harvey RJ. Defective escape behavior in DEAH-box RNA helicase mutants improved by restoring glycine receptor expression. J Neurosci (2013) 33(37):14638–44. doi: 10.1523/JNEUROSCI.1157-13.2013 PMC6705175 24027265

[15] Dong MB Wang G Chow RD Ye L Zhu L Dai X. Systematic immunotherapy target discovery using genome-scale in vivo CRISPR screens in CD8 T cells. Cell (2019) 178(5):1189–1204 e23. doi: 10.1016/j.cell.2019.07.044 31442407 PMC6719679

[16] Oza AM DiStefano MT Hemphill SE Cushman BJ Grant AR Siegert RK. Expert specification of the ACMG/AMP variant interpretation guidelines for genetic hearing loss. Hum Mutat (2018) 39(11):1593–613. doi: 10.1002/humu.23630 PMC6188673 30311386

[17] Wan Y Xue R Wang Y Zhang Q Huang S Wu W. The effect of neuropeptide y on brown-like adipocyte’s differentiation and activation. Peptides (2015) 63:126–33. doi: 10.1016/j.peptides.2014.10.018 25451330

[18] Wunsch L Holterhus PM Wessel L Hiort O. Patients with disorders of sex development (DSD) at risk of gonadal tumour development: Management based on laparoscopic biopsy and molecular diagnosis. BJU Int (2012) 110(11 Pt C):E958–65. doi: 10.1111/j.1464-410X.2012.11181.x 22540217

[19] Yang K Yang J Yi J. Nucleolar stress: Hallmarks, sensing mechanism and diseases. Cell Stress (2018) 2(6):125–40. doi: 10.15698/cst2018.06.139 PMC6551681 31225478

[20] Dannheisig DP Bachle J Tasic J Keil M Pfister AS. The wnt/beta-catenin pathway is activated as a novel nucleolar stress response. J Mol Biol (2021) 433(2):166719. doi: 10.1016/j.jmb.2020.11.018 33221336

[21] Fairman-Williams ME Guenther UP Jankowsky E. SF1 and SF2 helicases: family matters. Curr Opin Struct Biol (2010) 20(3):313–24. doi: 10.1016/j.sbi.2010.03.011 PMC2916977 20456941

[22] Tauchert MJ Fourmann JB Luhrmann R Ficner R. Structural insights into the mechanism of the DEAH-box RNA helicase Prp43. Elife (2017) 6. doi: 10.7554/eLife.21510 PMC5262380 28092261

[23] Maatouk DM DiNapoli L Alvers A Parker KL Taketo MM Capel B. Stabilization of beta-catenin in XY gonads causes male-to-female sex-reversal. Hum Mol Genet (2008) 17(19):2949–55. doi: 10.1093/hmg/ddn193 PMC2536503 18617533

[24] Rocha VB Guerra-Junior G Marques-de-Faria AP de Mello MP Maciel-Guerra AT. Complete gonadal dysgenesis in clinical practice: the 46,XY karyotype accounts for more than one third of cases. Fertil Steril (2011) 96(6):1431–4. doi: 10.1016/j.fertnstert.2011.09.009 21982289

[25] Lee PA Nordenstrom A Houk CP Ahmed SF Auchus R Baratz A. Global disorders of sex development update since 2006: Perceptions, approach and care. Horm Res Paediatr (2016) 85(3):158–80. doi: 10.1159/000442975 26820577

[26] Tanaka YO Mesaki N Kurosaki Y Nishida M Itai Y. Testicular feminization: role of MRI in diagnosing this rare male pseudohermaphroditism. J Comput Assist Tomogr (1998) 22(6):884–8. doi: 10.1097/00004728-199811000-00008 9843226

[27] Wisniewski AB Batista RL Costa EMF Finlayson C Sircili MH P Denes FT. Management of 46,XY Differences/Disorders of sex development (DSD) throughout life. Endocr Rev (2019) 40(6):1547–72. doi: 10.1210/er.2019-00049 31365064

[28] Loch Batista R Inacio M Prado Arnhold IJ Gomes NL Diniz Faria JA Rodrigues de Moraes D. Psychosexual aspects, effects of prenatal androgen exposure, and gender change in 46,XY disorders of sex development. J Clin Endocrinol Metab (2019) 104(4):1160–70. doi: 10.1210/jc.2018-01866 30388241

