# Supplementary figures and images for: A novel DEAH-box helicase 37 mutation associated with differences of sex development
Source: Front Endocrinol (Lausanne). 2023 Mar 30;14:1059159. doi: 10.3389/fendo.2023.1059159 (PMC10098359; doi:10.3389/fendo.2023.1059159)

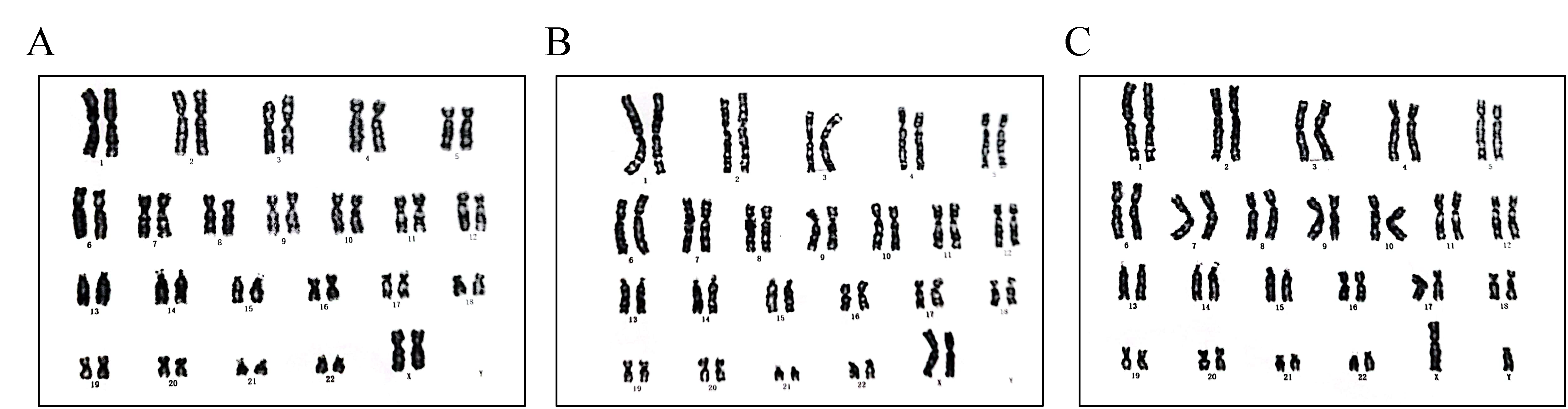

Supplement: Supplementary Figure 1 — Karyotype of III:3 (A), III:4 (B), and III:5 (C). [file Image_1.tiff]
